# GH, IGF-1, and Age Are Important Contributors to Thyroid Abnormalities in Patients with Acromegaly

**DOI:** 10.1155/2018/6546832

**Published:** 2018-01-15

**Authors:** Xia Wu, Lu Gao, Xiaopeng Guo, Qiang Wang, Zihao Wang, Wei Lian, Wei Liu, Jian Sun, Bing Xing

**Affiliations:** ^1^Department of Neurosurgery, Peking Union Medical College Hospital, Chinese Academy of Medical Sciences and Peking Union Medical College, Beijing 100730, China; ^2^China Pituitary Disease Registry Center, Chinese Pituitary Adenoma Cooperative Group, Beijing 100730, China; ^3^Key Laboratory of Endocrinology of National Health and Family Planning Commission, Beijing 100730, China; ^4^Department of General Surgery, Peking Union Medical College Hospital, Beijing 100730, China; ^5^Department of Pathology, Peking Union Medical College Hospital, Beijing 100730, China

## Abstract

**Purpose:**

To determine the prevalence, risk factors, and possible mechanisms of structural and endocrinological changes to the thyroid in acromegaly.

**Methods:**

We studied 93 acromegalic patients from PUMCH between January 2013 and December 2013. The demographic and clinical information were recorded. Specimens of pituitary adenomas and thyroid cancer were collected for BRAF mutation assessments.

**Results:**

Thyroid morphological abnormalities were found in 72 (77.4%) patients. Three (3.2%) were diagnosed with thyroid cancer. The thyroid gland volume was significantly increased in patients with higher random GH (*p* = 0.01), higher nadir GH (*p* = 0.008), and higher IGF-1 level (*p* = 0.018). Age (*p* = 0.002) was an independent risk factor for thyroid morphological abnormalities in acromegaly. The GH burden was significantly higher in patients with thyroid morphological abnormalities (*p* = 0.036). The BRAF V600E mutation was detected in the PTCs of the two patients with thyroid cancer.

**Conclusions:**

Both benign and malignant thyroid abnormalities are increased in the acromegalic population compared to those in the general population. Age at diagnosis is an independent risk factor for thyroid abnormalities, and GH burden may be a partial contributor. Early diagnosis, early treatment, and monitoring of postoperational endocrine levels are important for acromegalic patients.

## 1. Introduction

Acromegaly is a chronic disease that results from persistent hypersecretion of growth hormone (GH). Excess GH and subsequently elevated insulin-like growth factor-1 (IGF-1) have mitogenic and antiapoptotic properties. Thus, GH and IGF-1 are thought to be associated with tumorigenesis and cancer progression in patients with acromegaly. Previous studies have highlighted an increased risk for colorectal and thyroid cancers in patients with acromegaly [1, 2]. However, for many years, the prevalence of thyroid nodules and cancer in patients with acromegaly was variable between different studies [3]. The correlations between structural and endocrine alterations to the thyroid and patient demographic characteristics, GH levels, and IGF-1 levels in acromegaly remain controversial [4].

In addition, it is still not known whether thyroid cancer in acromegalic patients is associated with genetic events such as the BRAF V600E mutation. The incidence of thyroid disease in acromegaly probably varies between ethnic and geographical groups. Although China represents one-fifth of the world's population, the prevalence of this disease in China is unknown. Therefore, the aim of this study was to investigate the clinical characteristics and risk factors that were associated with thyroid structural, functional, and neoplastic alterations in acromegaly using the first case series in China.

## 2. Materials and Methods

### 2.1. Patients

Between January 2013 and December 2013, 146 patients who presented to the Department of Neurosurgery, Peking Union Medical College Hospital (PUMCH) were newly diagnosed with acromegaly [5]. The inclusion criteria were as follows: clinical features for acromegaly; GH levels that were greater than 1 ng/ml after an oral glucose tolerance test (OGTT) with 75 g of dextrose; elevated serum IGF-1 levels adjusted for gender and age; and a pituitary adenoma identified by contrast-enhanced magnetic resonance imaging (MRI). Exclusion criteria were as follows: pregnancy; a thyroid abnormality diagnosed before the onset of clinical symptoms of acromegaly, including thyroid nodules, thyroid cancer, and thyroid functional diseases; and previous thyroidectomy, radiotherapy, or medical therapy. In the end, 93 patients were enrolled in our study. This study was approved by the Ethical Committee of PUMCH. Informed consent was obtained from each patient.

### 2.2. Methods

Sex, age at diagnosis, and disease duration (determined from the time of the onset of symptoms to the time of inclusion into this study) were recorded for each patient. The serum GH (both random and nadir GH), IGF-1, free T4 (fT4), T4, free T3 (fT3), T3, and thyroid stimulating hormone (TSH) levels were determined using chemiluminescent immunometric assays (L2KGRH2, Siemens Healthcare Diagnostics Products Ltd., Glyn Rhonwy, Llanberis, Gwynedd LL55 4EL, UK). Random GH was defined as the fasting serum GH tested nearest to the time of diagnosis of acromegaly. Nadir GH was defined as the lowest serum GH during the OGTT. To estimate the cumulative level of patient exposure to GH, the disease duration multiplied by the random GH level, which was considered the GH burden, was recorded in this study. The reference values were 0.81–1.89 ng/dl for fT4, 4.30–12.50 *μ*g/dl for T4, 1.80–4.10 pg/ml for fT3, 0.66–1.92 ng/ml for T3, and 0.38–4.34 *μ*IU/ml for TSH.

Pituitary glands were scanned by contrast-enhanced MRI using a 3.0T MRI system (MAGNETOM Skyra, Siemens Healthcare, Erlangen, Germany). The volume of the pituitary adenoma was calculated using an elliptical model (*π*/6 multiplied by the height, width, and depth measured on MRI). Thyroid ultrasound was performed at the diagnosis of acromegaly by experienced specialists in ultrasound using a Philips iU22 ultrasound machine with high-frequency linear array transducers in the 8-to-15 MHz range. The characteristics of the thyroid, which included the number of nodules in the thyroid and the size, echogenicity, margins, internal content (presence of cystic lesions), shape, and vascular pattern of the thyroid, were thoroughly documented. The volume of the thyroid gland was calculated using the sum of the volume of each lobe and the isthmus based on an ellipsoid model. The diagnosis of thyroid structural changes was determined according to the thyroid imaging reporting and data system (TIRADS) for ultrasound features by Horvath in 2009 [6]. A partial or total thyroidectomy was performed in each patient who was classified as TIRADS category 5 (consistent with malignancy) per thyroid ultrasonographical examination. Thyroid cancer and pituitary adenoma specimens were available for each acromegalic patient who was pathologically diagnosed with thyroid cancer. The thyroid cancer and pituitary adenoma specimens of the same patient were evaluated for the BRAF gene mutation. DNA of collected tissues was extracted using the ReliaPrep™ FFPE gDNA Miniprep System kit (A2351, Promega) following the protocol provided by the manufacturer. Spectral absorbance of DNA was measured by a spectrophotometer (Merinton SMA4000). A validated, China Food and Drug Administration- (CFDA-) approved (State medical permit number 2013-3401388) diagnostic kit for V600E mutation of Human B-raf gene (RiQigen, Wuxi, China) based on amplification refractory mutation system (ARMS) was used for the detection of BRAF V600E mutation. For each sample, there was an external control assay and a mutation assay (in the same well). Each run contained a negative control and a positive control. The cycling conditions were as follows: denaturation for 2 minutes at 95°C; 5 seconds at 95°C, 30 seconds at 52°C, and 15 seconds at 72°C, repeated for 10 cycles (cycle 1); and 3 seconds at 93°C, 30 seconds at 56°C, and 30 seconds at 60°C, repeated for 35 cycles (cycle 2). Data was collected at 56°C cycle 2 (Agilent Technologies, Stratagene Mx3000P real-time PCR instrument). Run files were analyzed and interpreted based on the manual of the manufacturer.

### 2.3. Statistical Analysis

Categorical variables are presented as a number (percentage). Quantitative data are presented as the mean (±standard deviation) or median value (lower quartile, upper quartile). Comparisons between categorical variables were performed using the chi-square test. Comparisons between numerical variables were performed using the independent sample *t*-test and Mann–Whitney test. Multiple linear regression analysis was used to evaluate correlations among the clinical data. Binary logistic regression analysis was used to identify related risk factors. In all cases, a *p* value < 0.05 was considered statistically significant. All analyses were performed using SPSS statistics version 19.0 (SPSS Inc., Chicago, IL).

## 3. Results

### 3.1. Thyroid Structural and Neoplastic Alterations

Of the 93 patients included in our study, the mean age was 41.5 ± 12.9 years, with 40 (45.2%) males and 53 (54.8%) females. The average duration of the disease was 60 months (the 1st–3rd quartile, 24–96). Thyroid ultrasound was performed in all 93 patients. Seventy-two (77.4%) patients had structural changes to the thyroid, and only 21 (22.6%) patients had normal thyroids. Among the patients with thyroid morphological abnormalities, 42 (45.2%) had benign nodules, 20 (21.5%) had benign nodules together with goiter, and seven (7.5%) had diffuse goiter without nodules. Three patients (3.2%) were diagnosed with thyroid cancer, with one medullary thyroid carcinoma (MTC) and two papillary thyroid carcinomas (PTC) confirmed by surgical pathology. The patient with MTC was a 53-year-old female. She had a tumor size of 1.3 cm in the right lobe with metastatic involvement of the central (level VI) cervical lymph nodes (1 out of 2 lymph nodes). Immunohistochemistry revealed calcitonin (+), CgA (+), and Tg (−). One of the two patients with PTCs was a 66-year-old female. She had follicular variant of PTC with a tumor size of 0.5 cm in the left lobe, and another tumor size of 1.2 cm in the right lobe without lymph node metastasis. Immunohistochemistry revealed Ki-67 (index 2%) and VEGF (+). The other patient with PTC was a 41-year-old male. He had a conventional variant with a tumor size of 2.0 cm in the right lobe, with metastatic involvement of the central (level VI) cervical lymph nodes (1 out of 4 lymph nodes). He refused for immunohistochemistry staining.

### 3.2. Effect of GH and IGF-1 Levels on Thyroid Volume and Function

**Figure 1:**
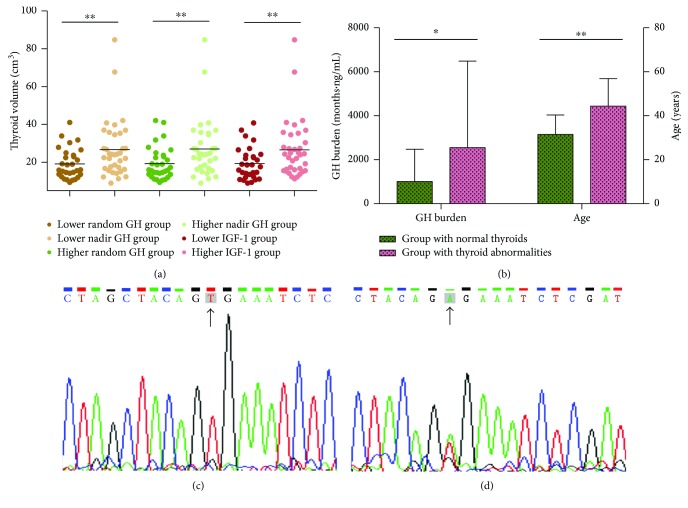
(a) Comparisons of thyroid volume in the groups divided by the median nadir GH, random GH, and IGF-1. ^∗^*p* value < 0.05; ^∗∗^*p* value < 0.01. (b) Comparisons of the GH burden and the age of diagnosis between the groups of patients with and without thyroid abnormalities. ^∗^*p* value < 0.05; ^∗∗^*p* value < 0.01. (c) Nucleotide sequencing result showing wild-type BRAF (indicated by an arrow) in the specimens of the pituitary adenoma of the patient with thyroid cancer. (d) Nucleotide sequencing result showing the T to A missense point mutation (indicated by an arrow) in the PTC of the patient with thyroid cancer.

Of the 93 patients included in our study, 86 (92.5%) completed measurements for the following hormone simultaneously: random and nadir GH, IGF-1, fT4, T4, fT3, T3, and TSH. Six (7.0%) were diagnosed with central hypothyroidism, while none of the 86 patients had primary hypothyroidism. Then, we divided these patients into two groups according to the median random GH value (13.7 ng/ml): group I (GH < 13.7 ng/ml) and group II (GH > 13.7 ng/ml). Unadjusted comparisons of demographical and clinical characteristics between the two groups are presented in Table 1. Similarly, two groups that were divided per median nadir GH value (9.10 ng/ml) and another two groups that were divided per median IGF-1 value (838.5 ng/ml) were analyzed using the same method (Table 1). Statistical analysis showed no difference between the two groups divided by the median random GH, nadir GH, and IGF-1 level with respect to sex and age. We found that the thyroid gland volume was significantly larger in group II with the higher random GH level (27.97 ± 16.16 versus 19.14 ± 7.98 cm^3^, *p* = 0.01), group II with the higher nadir GH level (28.41 ± 16.20 versus 19.31 ± 8.56 cm^3^, *p* = 0.008), and group II with the higher IGF-1 level (27.45 ± 16.13 versus 19.37 ± 8.46 cm^3^, *p* = 0.018) (Figure 1(a)). Multiple linear regression showed that after age, sex, and other possible predictors were considered, standardized coefficients were 0.395 (*p* = 0.002), 0.370 (*p* = 0.005), and 0.408 (*p* = 0.004) for the thyroid gland volume in the two groups classified by the median random GH level, nadir GH level, and IGF-1 level, respectively. Regarding thyroid endocrine function in the acromegalic patients, T3 (*p* = 0.01), T4 (*p* = 0.018), and fT3 (*p* = 0.004) significantly differed between the groups with the lower and higher IGF-1 levels. Although trends for lower TSH, higher T3, higher fT3, and higher fT4 were observed in the groups with higher random GH and higher nadir GH, no significant differences were observed for the following parameters: T3, T4, fT3, fT4, and TSH.

### 3.3. Risk Factors of Thyroid Abnormalities in Acromegalic Patients

Possible risk factors were compared between the group with normal thyroid glands and the group with structural changes to the thyroid (Table 2). The age was significantly higher in patients with thyroid abnormalities (44.38 ± 12.51 years versus 31.48 ± 8.87 years, *p* < 0.001). The sex, disease duration, pituitary adenoma volume, and thyroid gland volume were not significantly different between the patients with and without thyroid abnormalities. Regarding endocrine hormone levels, the GH burden was significantly higher in patients with thyroid abnormalities (1321.8 months ng/ml versus 463.8 months ng/ml, *p* = 0.036) (Figure 1(b)). No significant differences in the proportion of acromegalic patients with and without thyroid abnormalities were observed when patients were classified using the following parameters: random GH, nadir GH, and IGF-1 levels. By multivariate logistic regression analysis, age remained an independent risk factor for thyroid abnormalities in patients with acromegaly (OR = 1.092; 95% confidence interval (CI), 1.034–1.154; *p* = 0.002), while the GH burden lost its significance (OR = 1.000; 95% CI, 1.000–1.001; *p* = 0.090).

### 3.4. Genetic Results

The BRAF gene mutation was detected in the PTCs of two of the three patients who had been diagnosed with thyroid cancer (Figures 1(c) and 1(d)). Meanwhile, the BRAF mutation was not detected in any of the pituitary adenoma biopsies of the two patients. No BRAF mutation was found in either the thyroid or pituitary neoplasms of the patient diagnosed with MTC.

## 4. Discussion

Thyroid cancer is one of the most commonly detected cancers in acromegaly [7, 8]. Here, we determined the thyroid malignancy prevalence in a cohort of 93 Chinese patients with acromegaly to be 3.22%, which is higher than in the general population [2, 9]. Although the prevalence is comparable to that found in previous studies, it is near the lower limit of the reported rates of thyroid cancer from studies that were focused on other ethnicities (between 1.2% and 11%) [2, 10, 11]. Apart from the ethical and environmental differences, the relatively low prevalence of thyroid neoplasms in our study is probably an underestimation because thyroidectomy was performed only in patients classified as TIRADS category 5 through ultrasonography. Additionally, the difference in follow-up time may also contribute to the difference in the detection rate of thyroid malignancy. Consistent with previous reports, the excessive risk of thyroid malignancy in our study was confined to differentiated thyroid cancers, with PTC being the most common type [8, 12].

In our study, 77.4% of acromegalic patients exhibited thyroid abnormalities. The number was much higher than the reported prevalence in the general population [13]. Similar to the findings of our study in a Chinese population, a recent study of 205 Polish patients with acromegaly reported a prevalence rate for thyroid lesions at 77.6% [14]. Furthermore, thyroid structural changes among patients with acromegaly showed a higher prevalence in females than in males, which was consistent with the higher prevalence among women in the general population [15]. The most frequent thyroid abnormalities were benign nodules (66.7%), whose prevalence was twice as high as that in the general population. The rate was slightly higher than those reported in western countries (54% and 55.7%), partially because the Chinese population had excessive iodine intake, as mentioned above [16, 17].

Several explanations have been proposed for the increased rate of thyroid abnormalities in patients with acromegaly. Multiple studies have shown that a larger thyroid volume is associated with active acromegaly [14, 16]. Previously, in a study of 17 acromegalic patients carried out by Miyakawa et al., higher serum GH and IGF-1 had an analogous association with a larger thyroid volume [18]. Our study is the first to show that both the random GH and nadir GH levels, together with IGF-1, are positively correlated with thyroid volume in acromegaly. Thyroid cells can synthesize and secrete IGF-1 through GH stimulation; IGF-1 binding to IGF-1 receptors on thyroid cells activates autocrine signaling and consequently induces thyroid cell proliferation and activates antiapoptotic pathway in these cells [19, 20]. Regarding the nonautonomous thyroid changes in acromegaly, we are the first to show that IGF-1 has a significant influence on thyroid endocrine function, especially on serum T3. These data may indicate that in patients with acromegaly, IGF-1, rather than GH, mainly contributes to thyroid dysfunction. Therefore, it is important to monitor thyroid function in acromegalic patients with high IGF-1 levels. Previous studies have shown that GH modulates the activity of thyroxine deiodinase, leading to increased T3 and decreased reverse-T3 levels, though usually within normal range [21, 22]. Our study also showed no difference in thyroid endocrine function between the groups that were divided per nadir GH and per random GH.

A few studies have focused on the risks of developing thyroid abnormalities in acromegaly, though their conclusions remain controversial. Dagdelen et al. revealed that an older age at diagnosis is an important determinant of cancer development in acromegaly [2]. A Brazilian study of 106 acromegalic patients has shown that three factors, namely, female, older age, and a large thyroid volume, are significantly more common in patients with thyroid morphological abnormalities, while no differences are apparent for the disease duration, IGF-1, fT4, or TSH [23]. In slight contrast to previous studies, we found that the age at diagnosis was an independent risk factor for thyroid abnormalities in acromegaly, but thyroid endocrine levels, thyroid volume, and sex did not generate differences. This finding suggests that early diagnosis and early intervention are important for patients with acromegaly. Additionally, systematic thyroid examinations should be performed in acromegalic patients, especially older patients. Although the GH burden lost its significance in the multivariate logistic regression analysis, the GH burden was still significantly heavier in the group with thyroid abnormalities. These data suggest that long-term exposure to excessive GH plays an important role in the development of thyroid morphological abnormalities. Additionally, symptoms of acromegaly are often insidious, which produces a heavier GH burden. Therefore, early diagnosis is necessary for a lighter GH burden and a normal thyroid gland in acromegaly.

BRAF is a prominent oncogene that is commonly mutated in PTCs, and the mutation occurs in 45% of PTC cases on average [24]. The BRAF gene is usually mutated at the V600 position (V600E) and consequently activates the Ras-mitogen-activated protein kinase pathway. As summarized in Table 3, the mutation rate of BRAF V600E in the PTCs of patients with acromegaly varied in previous studies. A study by Aydin et al. compared the acromegalic and nonacromegalic patients with differentiated thyroid cancer (DTC), to find that acromegalic patients have a relatively low prevalence of BRAF V600E mutation [25]. Another study carried out by Kim et al. presented a similar result, which detected the BRAF mutation in the PTC sample of only 1 of 11 patients (9.1%) [26]. However, Mian et al. found that as many as seven acromegalic patients (70%) had the BRAF V600E mutation in the PTC samples [10]. Regarding pituitary adenomas, previous studies have detected low BRAF mutation rates [27, 28]. A study from Italy analyzed 50 patients with pituitary adenoma and found only one V600E mutation in a nonfunctioning pituitary adenoma sample [27]. To our knowledge, this study is the first to analyze the prevalence of the BRAF mutation in a pituitary adenoma and thyroid cancer from the same patient. In our samples, the most common oncotype was PTC (2 out of 3 cases) with a high frequency (2 out of 2 PTC samples) of the BRAF V600E mutation, whereas no mutation was detected in the pituitary adenoma biopsies of the same patients. The one case with MTC harbored no BRAF mutation either in the thyroid cancer or pituitary adenoma specimen; the BRAF mutation occurred in 66.7% of thyroid cancer samples but was not detected in the pituitary adenoma samples from the acromegaly patients. Though the limited number of thyroid neoplasms in this study prevents us from drawing conclusions regarding the correlation between the BRAF mutation and thyroid cancer in acromegaly, it is likely that the BRAF V600E mutation occurs often in the PTCs of acromegalic patients. Further studies with a larger number of patients and long-term follow-up are needed to understand the role of BRAF in thyroid abnormalities in acromegaly.

The authors of a recent systemic review of this topic have summarized the main reported findings for thyroid diseases in acromegaly. We noted that none of the main studies originated from China. To our knowledge, this study is the first and largest performed in China to address thyroid diseases. Moreover, our study systematically analyzed thyroid morphological and endocrine changes, along with the risk factors and possible mechanisms of these changes in acromegaly.

This study has some limitations, such as the lack of a control group and being retrospective. Another limitation was that the patients were submitted to thyroid surgery based on TIRADS classification instead of cytology, and, therefore, the prevalence of thyroid cancer may be underestimated.

In conclusion, our study confirms the high frequency of both benign and malignant thyroid abnormalities in Chinese acromegalic patients. The age at diagnosis is an independent risk factor for thyroid morphological abnormalities. The GH burden may also be a partial contributor. Therefore, early diagnosis and early treatment are important for acromegalic patients. Furthermore, the IGF-1 level has a significant influence on thyroid volume and thyroid hormone levels in acromegaly, which indicates that postoperational endocrine levels are worth closely monitoring for a good acromegaly prognosis.

## Figures and Tables

**Table 1:** Comparisons of demographical and clinical characteristics between groups divided by median random GH, nadir GH, and IGF-1.

	Random GH (*n* = 86)			Nadir GH (*n* = 86)			IGF-1 (*n* = 86)		
	Group I	Group II	*p* value	Group I	Group II	*p* value	Group I	Group II	*p* value
Sex (*n*, %)									
Male (*n*)	20 (46.5)	20 (46.5)	0.369	19 (44.2)	21 (48.8)	0.665	10 (23.3)	14 (32.6)	0.336
Female (*n*)	23 (53.5)	23 (53.5)		24 (55.8)	22 (51.2)		33 (76.7)	29 (67.4)	
Age (years)	39.53 ± 12.70	41.58 ± 12.27	0.449	39.07 ± 12.91	42.05 ± 11.95	0.27	40.37 ± 12.71	40.16 ± 12.48	0.939
Thyroid volume (cm^3^)	19.14 ± 7.98	27.97 ± 16.16	**0.010** ^∗^	19.31 ± 8.56	28.41 ± 16.20	**0.008** ^∗∗^	19.37 ± 8.46	27.45 ± 16.13	**0.018** ^∗^
(Multiple linear regression)	*β* = 0.395		**0.002** ^∗∗^	*β* = 0.370		**0.005** ^∗∗^	*β* = 0.408		**0.004** ^∗∗^
TSH (*μ*IU/ml)	1.52 ± 0.92	1.43 ± 1.44	0.743	1.53 ± 0.92	1.43 ± 1.44	0.711	1.66 ± 1.38	1.34 ± 1.02	0.234
T3 (ng/ml)	0.96 ± 0.31	1.04 ± 0.31	0.244	0.98 ± 0.31	1.02 ± 0.32	0.592	0.91 ± 0.28	1.09 ± 0.31	**0.010** ^∗^
(Multiple linear regression)							*β* = 0.254		**0.044** ^∗^
T4 (*μ*g/dl)	8.77 ± 1.99	8.86 ± 2.08	0.847	8.81 ± 1.97	8.82 ± 2.11	0.978	8.25 ± 1.03	9.32 ± 1.89	**0.018** ^∗^
FT3 (pg/ml)	2.89 ± 0.68	2.85 ± 0.66	0.256	2.91 ± 0.64	2.82 ± 0.70	0.559	2.63 ± 0.59	3.06 ± 0.72	**0.004** ^∗∗^
FT4 (ng/dl)	1.21 ± 0.25	1.15 ± 0.20	0.232	1.22 ± 0.25	1.13 ± 0.20	0.108	1.15 ± 0.26	1.20 ± 0.18	0.272

^∗^ *p* values less than 0.05; ^∗∗^*p* values less than 0.01.

**Table 2:** Risk factors of thyroid abnormalities in patients with acromegaly.

	Patients with normal thyroid (*n* = 72)	Patients with thyroid abnormalities (*n* = 21)	*p* value	Binary logistic regression analysis	
				OR (95% CI)	*p* value
Sex (*n*, %)					
Male	28 (38.9)	12 (57.1)	0.137	0.628 (0.198–1.992)	0.249
Female	44 (61.1)	9 (42.9)			
Age (years)	44.38 ± 12.51	31.48 ± 8.87	**<0.001** ^∗∗^	1.092 (1.034–1.154)	**0.002** ^∗∗^
Acromegaly duration (months)	60 (20, 114)	36 (18, 66)	0.089	1.002 (0.992–1.013)	0.661
Pituitary adenoma volume (cm^3^)	1.15 (0.34, 2.66)	2.26 (0.71, 5.68)	0.223	1.027 (0.810–1.301)	0.828
Thyroid volume (cm^3^)	24.32 ± 14.16	20.36 ± 8.56	0.284	1.028 (0.959–1.102)	0.432
BMI (kg/m^2^)	27.08 ± 3.94	25.56 ± 4.16	0.136	1.122 (0.944–1.335)	0.192
TSH (*μ*IU/ml)	1.63 ± 1.47	1.36 ± 0.93	0.433	N/A	N/A
T3 (ng/ml)	0.99 ± 0.31	1.02 ± 0.29	0.781	N/A	N/A
T4 (*μ*g/dl)	8.86 ± 2.07	8.33 ± 1.76	0.303	N/A	N/A
Random GH (ng/ml)	15.25 (8.60, 52.50)	12.0 (7.30, 33.95)	0.212	1.013 (0.996–1.029)	0.127
Nadir GH (ng/ml)	10.45 (5.70, 38.30)	7.04 (4.94, 22.03)	0.194	1.014 (0.996–1.032)	0.135
IGF-1 (ng/ml)	849.65 ± 310.01	822.16 ± 214.02	0.719	1.001 (0.999–1.003)	0.393
GH burden (months ng/ml)	1321.8 (313.2, 3360.0)	463.8 (187.2, 1180.0)	**0.036** ^∗^	1.000 (1.000–1.001)	0.090

^∗^ *p* values less than 0.05; ^∗∗^*p* values less than 0.01.

**Table 3:** General characteristics of BRAF mutation in DTCs of patients with acromegaly.

Study	Year	Country	Patients with acromegaly	Number of DTCs	Types of DTCs	BRAF mutation rate
Kim et al. [26]	2014	Korea	60	15 (25.0%)	15 PTCs	1/11 (9.1%)
Mian et al. [10]	2014	Italy	113	12 (10.6%)	10 PTCs and 2 FTCs	7/12 (58.3%)
Aydin et al. [25]	2016	Turkey	NA	14	11 PTCs and 3 other types^∗^	2/14 (14.3%)
Present study	2017	China	93	3 (3.2%)	2 PTCs and 1 MTC	2/3 (66.7%)

DTC: differentiated thyroid cancer; PTC: papillary thyroid cancer; FTC: follicular thyroid cancer; MTC: medullary thyroid cancer; NA: not available. ^∗^Other types included two well-differentiated tumors with undetermined follicular neoplasms and one follicular variant and follicular neoplasms.
